# Rationale, design and baseline characteristics of REMODEL, a mechanism-of-action trial with semaglutide in people with type 2 diabetes and chronic kidney disease

**DOI:** 10.1093/ndt/gfaf114

**Published:** 2025-07-03

**Authors:** David Z I Cherney, Nicolas Belmar, Petter Bjornstad, Milenta M Chacko, Thomas P Gunnarsson, Jeffrey B Hodgin, Matthias Kretzler, Menno Pruijm, Philip A Schytz, Katherine R Tuttle

**Affiliations:** Department of Medicine, Division of Nephrology, University Health Network, Toronto, Ontario, Canada; Novo Nordisk A/S, Søborg, Denmark; Division of Metabolism, Endocrinology and Nutrition, Department of Medicine, and Division of Endocrinology, Department of Pediatrics, University of Washington, Seattle, WA, USA; Division of Renal Diseases and Hypertension, Department of Medicine, Section of Pediatric Endocrinology, Department of Pediatrics, University of Colorado Anschutz Medical Campus, Aurora, CO, USA; Novo Nordisk GBS India, Bangalore, India; Novo Nordisk A/S, Søborg, Denmark; Department of Pathology and Clinical Laboratories, University of Michigan, Ann Arbor, MI, USA; Department of Internal Medicine and Bioinformatics, University of Michigan, Ann Arbor, MI, USA; Service of Nephrology, University Hospital of Lausanne and University of Lausanne, Lausanne, Switzerland; Novo Nordisk A/S, Søborg, Denmark; Providence Medical Research Center, Providence Inland Northwest Health, Spokane WA, USA; Kidney Research Institute and Nephrology Division, University of Washington School of Medicine, Seattle, WA, USA

**Keywords:** functional magnetic resonance imaging, gene expression, incretin therapy, kidney, nutrient-stimulated hormone (NuSH) therapy

## Abstract

**Background:**

Type 2 diabetes (T2D) is the leading cause of chronic kidney disease (CKD) and kidney failure globally. Semaglutide, a glucagon-like peptide-1 (GLP-1) receptor agonist, reduces the risk of major kidney, cardiovascular and mortality outcomes in people with T2D and CKD, but the mechanism of action remains unclear.

**Methods:**

The REMODEL trial (NCT04865770) is a 52-week placebo-controlled, double-blind, parallel-group, randomized trial in adults with T2D and CKD. Inclusion criteria include haemoglobin A1c (HbA1c) ≤9%, estimated glomerular filtration rate (eGFR) ≥30–≤75 ml/min/1.73 m^2^ and urine albumin:creatinine ratio (UACR) ≥20–<5000 mg/g. The co-primary outcomes were magnetic resonance imaging (MRI) based, including change in kidney oxygenation, perfusion and inflammation. Secondary outcomes include change from baseline in creatine clearance rate, urinary sodium excretion, albumin excretion rate and kidney fibrosis and blood flow parameters measured by MRI. A subgroup had kidney biopsies at baseline and at the end of the treatment for tissue-based interrogation, including single nucleus and spatial transcriptomics, pathology and advanced histological assessment.

**Results:**

Across eight countries, 106 participants (*n* = 33, biopsy subgroup) were enrolled. The mean age was 65.3 years [standard deviation (SD) 9.9] at baseline with HbA1c of 7.1% (SD 0.9), creatinine-based eGFR of 51.1 ml/min/1.73 m^2^ (SD 10.4) and median UACR of 187.3 mg/g (interquartile range 60.5–546.4). Renin–angiotensin system inhibitor use was 98.1% and sodium–glucose co-transporter 2 inhibitor use was 38.7%. In the kidney biopsy subgroup, baseline characteristics were like those of the full population. Histological analysis of kidney tissues revealed 17 participants with primarily diabetic nephropathy, 6 participants with primarily vascular features, 9 with mixed diabetic nephropathy and vascular characteristics and 1 with membranous nephropathy.

**Conclusion:**

The REMODEL trial leverages multipronged approaches to investigate the kidney-specific effects and underlying mechanisms of semaglutide in a representative population of people with T2D and CKD, which supports the generalizability and clinical relevance of the findings.

## Graphical Abstract

**Graphical Abstract:**
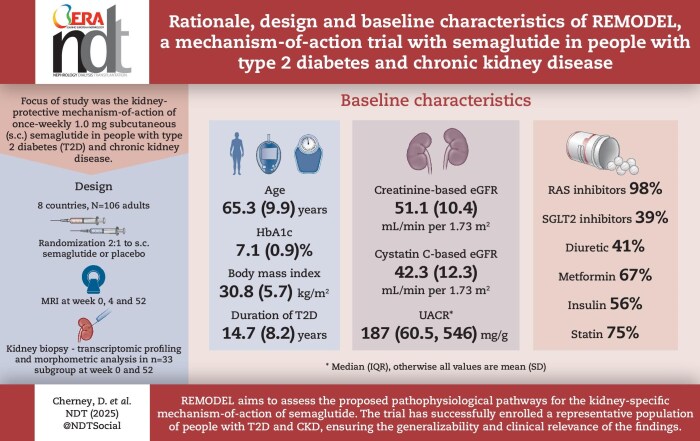

KEY LEARNING POINTS **What was known:**

- Semaglutide, a glucagon-like peptide-1 (GLP-1) receptor agonist, reduces risks of major kidney, cardiovascular and mortality outcomes in people with type 2 diabetes (T2D) and chronic kidney disease (CKD), as highlighted in the recent FLOW trial, but the mechanism of action (MoA) remains unclear.

**This study adds:**

- REMODEL trial (NCT04865770) is a 52-week phase 3b placebo-controlled clinical trial investigating the potential MoA of subcutaneous semaglutide at a dose of 1.0 mg/week in people with T2D and CKD.
- The REMODEL trial will comprehensively investigate several proposed kidney-specific MoAs for semaglutide by applying advanced methods such as functional kidney magnetic resonance imaging and cell type–specific gene expression analyses from kidney biopsies.
- The REMODEL trial has successfully enrolled a representative population of people with T2D and CKD, which will support the real-world applicability and generalizability of the trial results.

**Potential impact:**

- The REMODEL trial aims to identify the key MoA of semaglutide in people with T2D and CKD by elucidating the physiological, cellular and molecular pathways in the context of treatment responses.
- Scientific evidence will be generated to lay a foundation for precision-guided use of semaglutide for CKD in people with T2D.
- The REMODEL trial may provide important insights into the MoA of semaglutide that are distinct from other kidney-protective drug classes such as sodium–glucose co-transporter 2 inhibitors and non-steroidal mineralocorticoid receptor antagonists.

## INTRODUCTION

Chronic kidney disease (CKD) is a major health burden globally, with an estimated prevalence of 9.1% [1, 2]. CKD contributes substantially to kidney failure, cardiovascular disease (CVD) and overall morbidity and mortality worldwide [1, 2]. Type 2 diabetes (T2D) is a well-established contributor to the development of CKD, with approximately half of all cases of kidney failure occurring in persons with T2D [3, 4]. In addition, there is growing interest in the interplay among CKD, CVD and T2D, termed cardiovascular–kidney–metabolic (CKM) syndrome, with a significant proportion of diabetes-associated excess CVD risk occurring in people with CKD [5–7]. Mechanistically, the progression of CKD is complex, especially when considering comorbidities such as T2D and CVD. Several factors, including hypoxia, inflammation, mitochondrial dysfunction, oxidative stress and haemodynamic changes, all contribute to disease progression, ultimately leading to fibrosis and kidney failure [5–7]. Given this complex molecular landscape, in-depth mechanistic studies of new therapies are important. These studies are essential to understand how treatment improves kidney outcomes and to facilitate a precision medicine approach to treatment. Moreover, elucidating these mechanisms aids future drug development by identifying novel targets and pathways, ultimately advancing our ability to treat CKD effectively [8].

Current guidelines support the use of renin–angiotensin system (RAS) inhibitors and sodium–glucose co-transporter 2 inhibitors (SGLT2is) to slow the progression of CKD, leading to improved CKM outcomes [9–11]. In addition, the non-steroidal mineralocorticoid receptor agonist finerenone reduces the risk of clinically important cardiovascular and kidney outcomes in people with T2D and CKD [12]. Recently, glucagon-like peptide-1 receptor agonists (GLP-1RAs) have become a recommended therapy for people with T2D and CKD and are recommended as a risk-based treatment to improve glucose control, weight loss and cardiovascular risk management in this population [11–13]. This approach has been bolstered by the results from the FLOW trial (NCT03819153) in people with T2D and CKD, which showed that semaglutide, a GLP-1RA, versus placebo produced a 24% relative risk reduction in the primary outcome, a composite of kidney failure, a ≥50% reduction in estimated glomerular filtration rate (eGFR) from baseline or death from cardiovascular or kidney-related causes [13]. However, the underlying mechanism of action (MoA) of kidney protection with semaglutide remains elusive.

**Figure 1:**
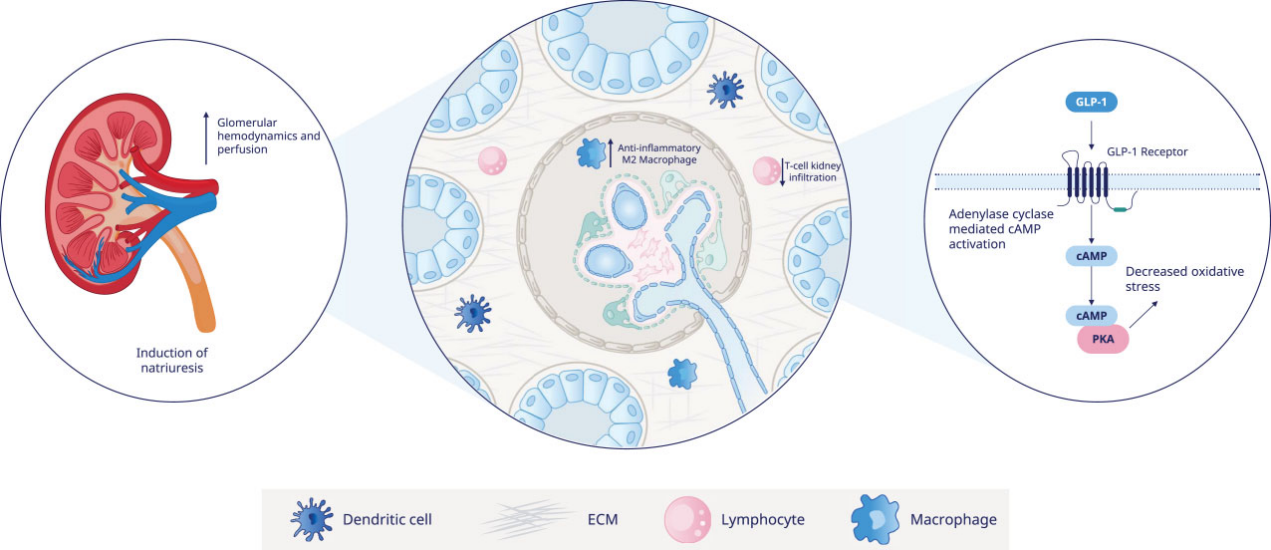
Proposed mechanisms by which GLP-1RAs may have direct effects on the kidney in people with T2D and CKD. ECM: extracellular matrix; PKA: protein kinase A.

GLP-1RAs reduce CKD risks only partially via indirect mechanisms such as improved glycaemic control, systolic blood pressure reduction and weight loss, implicating direct protective mechanisms in the kidney [14–17]. Several direct MoAs have been proposed for GLP-1R-mediated kidney protection in CKD, including suppression of kidney inflammation and oxidative stress, induction of natriuresis and improved glomerular haemodynamics and perfusion (Fig. 1). Although incompletely understood, GLP-1R expression in the kidney has been reported to be mainly in the smooth muscle cells of the preglomerular vasculature, where receptor activation can induce vasodilation [18]. GLP-1R expression is also reported in T cells, where activation of the receptor inhibits T cell proliferation and could reduce inflammation and oxidative stress by systemic or intrakidney T cells [19–21]. In addition, there is growing evidence that GLP-1R activation can influence macrophage lineage by promoting an M2 anti-inflammatory macrophage population in the kidney [22, 23]. Moreover, GLP-1RA therapy has been shown to reduce the systemic markers of inflammation in humans, with semaglutide exposure being associated with reduced expression of multiple inflammatory genes in different mouse models [24, 25]. GLP-1R activation may also reduce cellular oxidative stress, potentially through a cyclic adenosine monophosphate (cAMP)–protein kinase A–mediated pathway [26–28]. Finally, GLP-1 has been shown to support kidney oxygenation in healthy volunteers through two mechanisms: inducing natriuresis (which attenuates oxygen demand) and increasing cortical and medullary perfusion, as assessed by magnetic resonance imaging (MRI) [16, 29, 30]. Kidney oxygenation can be measured using blood oxygenation level–dependent (BOLD) MRI, a technique that estimates renal tissue oxygenation based on paramagnetic properties of deoxyhaemoglobin. Deoxyhaemoglobin creates local magnetic susceptibility gradients, which are measured to infer tissue oxygenation levels, assuming the blood deoxyhaemoglobin levels correlate inversely with partial pressure of oxygen levels in local tissue [31]. Taken together, these results are consistent with a direct GLP-1 role in kidney protection, but whether similar changes occur in people with T2D and CKD is not fully understood.

To gain comprehensive insight into the mechanisms of GLP-1RAs in CKD, a multifaceted approach leveraging recent technological advances to understand molecular MoAs in their cellular and structural context is needed. For example, a single-cell RNA sequencing study in kidney biopsies of people with youth-onset T2D gave an in-depth molecular analysis of the kidney-protective mechanisms and metabolic reprogramming seen with SGLT2i use [32]. Coupling this molecular approach with comprehensive clinical phenotyping and advanced non-invasive MRI techniques will yield new information about the effects of GLP-1RA on the human kidney.

The REMODEL trial (NCT04865770) will assess multiparametric MRI-based measures of changes in kidney oxygenation, global kidney perfusion, inflammation and fibrosis as well as multiple clinical parameters. In addition, a subgroup of participants will undergo sequential kidney biopsies both at baseline and at the end of treatment for tissue-based interrogation including single-nucleus RNA sequencing (snRNA-seq) and spatial transcriptomic profiling, pathology and morphometric examination. In this article we describe the study design and report the currently available clinical and histological baseline characteristics of the REMODEL population. The primary and secondary outcomes (including MRI and multi-omics parameters) will be presented once the trial is completed.

## MATERIALS AND METHODS

### Trial design and conduct

The REMODEL trial is a 52-week, multinational, randomized, double-blind, parallel-group, placebo-controlled phase 3b trial comparing subcutaneous semaglutide (1.0 mg/week) with a matched placebo. Both randomized groups received the standard of care at the time the trial began (2021), including RAS inhibition. SGLT2i use at randomization was capped at 50% in the full trial population, with stratification based on SGLT2i use employed to ensure representative use between both treatment groups. Use of other GLP-1RAs or finerenone was not permitted in trial participants at randomization or during the trial and SGLT2i use was not permitted to be initiated after randomization. The 1.0-mg once-weekly dose of semaglutide was selected to align with approved doses for treatment of hyperglycaemia, as in the FLOW and the SUSTAIN 6 (NCT01720446) trials [13, 33].

**Figure 2:**
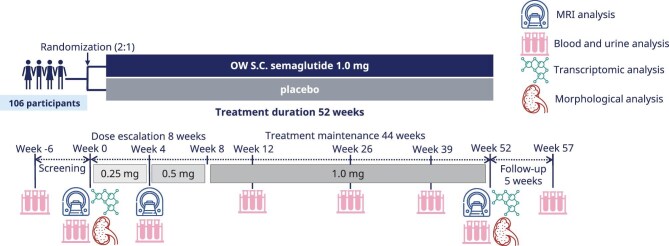
Summary of the REMODEL trial design. OW: once weekly; s.c.: subcutaneous.

Eligible participants were randomized in a 2:1 ratio to semaglutide or placebo. The trial period was 52 weeks with a follow-up period of 5 weeks (Fig. 2). An 8-week dose escalation regimen was employed at the start of treatment, with dose escalation from 0.25 mg/week for 4 weeks to 0.5 mg/week for 4 weeks as tolerated, followed by a maintenance dose of 1.0 mg/week throughout the remainder of the treatment period. The trial included a subgroup undergoing kidney biopsies at baseline and the end of treatment.

The trial was conducted in accordance with the International Council on Harmonization of Technical Requirements for Registration of Pharmaceuticals for Human Use Good Clinical Practice guidelines and the Declaration of Helsinki. The trial protocol was approved by the institutional review board and ethics committee at each participating centre. All participants provided written informed consent before any trial-related activity. The trial was sponsored by Novo Nordisk.

Eligible individuals fulfilled the key inclusion and exclusion criteria (Table 1). These eligibility criteria were designed to enrol a representative population of individuals with both T2D and CKD to produce generalizable and clinically relevant findings.

**Table 1:** Key inclusion and exclusion criteria for all participants and the biopsy subgroup.

Analysis group	Inclusion criteria	Exclusion criteria
Full population (*N* = 106)	• Age ≥18 years at the time of signing informed consent • Diagnosed with T2D ≥180 days prior to the day of screening • HbA1c ≤9.0% (≤75 mmol/mol) • RAS inhibitor use in the form of ACE inhibition or an ARB (treatment dose stable for 28 days) • For participants not in the biopsy subgroup: serum creatinine–based eGFR ≥30–≤75 ml/min/1.73 m^2^^a,b^ • UACR ≥20–<5000 mg/g	• Known or suspected hypersensitivity to trial or related products • Female who is pregnant, breastfeeding or intends to become pregnant or is of child-bearing potential and not using a highly effective contraceptive method • Receipt of any investigational medicinal product within 60 days before the day of screening • Any disorder that in the investigator's opinion might jeopardize participants safety or compliance with the protocol • MI, stroke, hospitalization for unstable angina or TIA within 180 days prior to the day of screening; ≥NYHA class III or planned coronary, carotid or peripheral artery revascularization • Presence or history of malignant neoplasms (other than basal or squamous cell skin cancer, *in situ* carcinomas of the cervix or *in situ* prostate cancer) within 5 years prior to the day of screening • Congenital or hereditary kidney diseases including polycystic kidney disease or autoimmune kidney diseases including glomerulonephritis or congenital urinary tract • Use of any GLP-1RA within 30 days prior to screening • Use of finerenone within 30 days prior to screening • Personal or first-degree relative(s) history of multiple endocrine neoplasia type 2 or medullary thyroid carcinoma • Chronic or intermittent haemodialysis or peritoneal dialysis • Uncontrolled and potentially unstable diabetic retinopathy or maculopathy^d^ • Treatment with systemic anti-inflammatory or immunosuppressant drugs within 90 days prior to screening • Inadequately treated blood pressure, defined as systolic ≥180 mmHg or diastolic ≥110 mmHg at screening • Any contraindication for MRI according to the standard checklist used in clinical routine • Abdominal height in the supine position >35 cm, measured as the vertical distance from the back to the tallest point on the abdomen while the participant is lying flat on his/her back • A prior solid organ transplant or awaiting a solid organ transplant • Combination use of ACE inhibitor and ARB • Depending on baseline SGLT2i use: For subjects not treated with an SGLT2i at baseline, treatment with an SGLT2i within 90 days of screening; for participants treated with an SGLT2i at baseline, stable dose of SGLT2i for <90 days prior to screening
Biopsy subgroup (*n* = 33)	In addition to the above: • Serum creatinine–based eGFR ≥40–≤75 ml/min/1.73 m^2^^a,b,c^ • Fulfil all biochemical, imaging and clinical requirements on the day of biopsy • Kidney cortex ≥1 cm (both kidneys) • Kidney length ≥8 cm (both kidneys)	In addition to the above: • Any condition with a single kidney • Evidence of a bleeding disorder • Any anti-platelet agent use or use of other drugs that can be associated with bleeding that cannot be safely stopped prior to and after the biopsy • Any NSAIDs or were chronic anticoagulant users up to 7 days before screening • An allergy to iodinated contrast if using computed tomography–guided kidney biopsy procedures • Unwilling to receive a blood transfusion (if needed)

a Creatinine-based eGFR was calculated per the CKD-EPI 2009 equation [40].

b At the time of sample collection, the subject must be in usual health condition as evaluated by the investigator.

c A higher eGFR was selected in the biopsy subgroup compared with all participants to reduce risk of bleeding in accordance with guidance at the time of starting the trial. Verified by a fundus examination performed within the past 90 days prior to screening or in the period between screening (week −6) and baseline (week 0). Pharmacological pupil dilation is a requirement unless using a digital fundus photography camera specified for non-dilated examination.

d Including claustrophobia or metallic foreign bodies, metallic implants, internal electrical devices or permanent makeup/tattoos that cannot be declared MR compatible.

ACE: angiotensin-converting enzyme; ARB: angiotensin receptor blocker; MI: myocardial infarction; NSAID: non-steroidal anti-inflammatory drug; NYHA: New York Heart Association; TIA: transient ischaemic attack.

### Biopsy subgroup

#### Morphologic and morphometric analysis

Kidney biopsies were performed in a subgroup of participants. A total of 33 baseline biopsies were referred to the Renal Pathology Division at the University of Michigan (Ann Arbor, MI, USA) for morphologic and morphometric assessment of kidney tissue using light and electron microscopy. The 3-μm-thick sections of formalin-fixed tissue were sectioned and stained with haematoxylin and eosin, periodic acid–Schiff, Jones silver methenamine and Masson's trichrome for light microscopic examination. Slides were scanned to whole slide images and quantitative assessment of glomerular tuft and mesangial area were performed using QuPath software [34]. The mesangial index was calculated as a percentage of mesangial area per glomerular tuft area. Glutaraldehyde-fixed samples for electron microscopy were processed according to standard methods and digital images were obtained for glomerular basement membrane (GBM) measurements. GBM thickness was determined by the orthogonal intercept method [35]. Arteriosclerosis was scored according to the 2018 Banff classification [36]. The biopsy samples were categorized into three endophenotypes (diabetic nephropathy, vascular and mixed) as previously described [37]. Briefly, the diabetic nephropathy endophenotype featured glomerular hypertrophy, diffuse or nodular mesangial expansion and GBM thickening, the vascular endophenotype featured ischaemic-appearing glomeruli, arteriosclerosis, interstitial fibrosis and tubular atrophy and the mixed endophenotype featured a combination of both the diabetic nephropathy and vascular endophenotypes.

#### Outcome measures

The primary objective of the REMODEL trial was to investigate the effect of semaglutide versus placebo at 52 weeks on kidney oxygenation, perfusion and inflammation, as measured by MRI in participants with T2D and CKD. To achieve the primary objective, multiparametric MRI-based co-primary outcomes were defined, including change from baseline in cortical and medullary kidney oxygenation as assessed by BOLD MRI (R2*), change from baseline in global kidney perfusion assessed by phase contrast MRI and change from baseline in cortical and medullary inflammation and microstructure assessed by MRI T1 mapping [31, 38, 39]. The co-primary outcomes were chosen as kidney oxygenation, perfusion and inflammation are closely intertwined and measured simultaneously in each MRI scan in the REMODEL trial. Supportive secondary outcomes included change from baseline in creatine clearance rate, urinary sodium excretion, albumin excretion rate, kidney fibrosis and blood flow parameters by MRI (apparent diffusion coefficient and renal artery resistive index). For eGFR calculation, the Chronic Kidney Disease Epidemiology Collaboration (CKD-EPI) 2009 formula was used based on serum creatinine and cystatin C levels [40]. Changes in genome-wide steady-state gene expression profiles will be evaluated in the kidney biopsies using a combination of snRNA-seq and spatial transcriptomic technologies, with specific focus on inflammatory and oxidative stress–related gene expression. Additionally, changes in GBM width will serve as supportive secondary outcomes in the kidney biopsy subgroup. Exploratory outcomes will include additional morphometric assessments from kidney biopsies, additional sequences from MRIs as well as inflammatory and oxidative stress biomarkers in blood and urine (Supplementary Table S1). Safety data from the full population were collected, including adverse events (AEs) and serious adverse events (SAEs). In the kidney biopsy subgroup, procedural safety was assessed by the development of bleeding, obstructive or infectious complications.

#### Statistics

The REMODEL trial is explorative and no confirmatory hypothesis testing was planned. An analysis of covariance model will be utilised to estimate the treatment difference between semaglutide and placebo at week 52 (compared with baseline), using actual treatment, region and stratification factors [SGLT2is at baseline (yes/no) and MRI field strength (1.5/3.0 T)] as categorical effects and the baseline endpoint value as a covariate. The estimated treatment difference between semaglutide and placebo will be presented together with the associated two-sided 95% confidence interval (CI) and *P*-value. Analyses and hypothesis-generating *P*-values from statistical models are considered descriptive in nature. No adjustment for multiple testing will be performed except separately in connection with analyses of gene expression data, where methods for controlling false discovery rates will be applied as needed. Missing data, either truly missing or omitted from analysis as per the estimand strategy, will be addressed using multiple imputations, assuming data are missing at random, and will be derived from the same treatment group.

Although the REMODEL trial has co-primary outcomes, the sample size was solely determined by the expected semaglutide-induced changes in oxygenation as measured with BOLD MRI (R2*). BOLD MRI (R2*) was used due to the lack of treatment studies using phase contrast and T1 MRI mapping. The REMODEL trial is designed to have at least 80% marginal power to detect a treatment difference in kidney oxygenation of a 10% change from baseline after 52 weeks as measured by BOLD MRI (R2*) in the cortex and medulla, respectively.

## RESULTS

### Participant demographics and baseline characteristics (full population and biopsy subgroup)

In total, 106 participants were enrolled across eight countries (Canada, Denmark, France, Italy, Poland, Spain, the USA and South Africa) with 33 kidney biopsy subgroup participants from five countries (Denmark, France, Poland, Spain and the USA). Participants in the full population had a mean age of 65.3 years [standard deviation (SD) 9.9], 76.4% were men and 61.3% identified as White (Table 2). Similarly, participants in the biopsy subgroup had a mean age of 65.4 years (SD 10.3), 87.9% were men and 54.5% identified as White. The mean haemoglobin A1c (HbA1c) was similar in the full population [7.1% (SD 0.9)] and the biopsy subgroup [7.1% (SD 1.0)]; 45.3% and 45.5% of the full population and biopsy subgroup had an HbA1c <7%, respectively. The mean baseline duration of T2D was 14.7 years (SD 8.2) and 15.3 years (SD 8.4), body mass index (BMI) was 30.8 kg/m^2^ (SD 5.7) and 30.7 kg/m^2^ (SD 6.4) and body weight was 89.6 kg (SD 18.9) and 92.9 kg (SD 15.2) at baseline for the full population and the biopsy subgroup, respectively.

**Table 2:** Baseline demographics and characteristics.

Variable	Full population (*n* = 106)	Biopsy subgroup (*n* = 33)
Age (years), mean (SD)	65.3 (9.9)	65.4 (10.3)
Men, *n* (%)	81 (76.4)	29 (87.9)
Race, *n* (%):		
White	65 (61.3)	18 (54.5)
Asian	15 (14.2)	5 (15.2)
Black or African American	10 (9.4)	4 (12.1)
American Indian or Alaska Native	2 (1.9)	1 (3.0)
Other	4 (3.8)	1 (3.0)
Not reported	10 (9.4)	4 (12.1)
Ethnicity, *n* (%):		
Hispanic or Latino	8 (7.5)	2 (6.1)
Not Hispanic or Latino	87 (82.1)	26 (78.8)
Not reported	11 (10.4)	5 (15.2)
Smoking status, *n* (%)		
Current smoker	15 (14.2)	6 (18.2)
Previous Smoker	35 (33.0)	13 (39.4)
Never smoked	56 (52.8)	14 (42.4)
Body weight (kg), mean (SD)	89.6 (18.9)^b^	92.9 (15.2)
BMI (kg/m^2^), mean (SD)	30.8 (5.7)^b^	30.7 (6.4)
HbA1c (%), mean (SD)	7.1 (0.9)	7.1 (1.0)
HbA1c <7%, *n* (%)	48 (45.3)	15 (45.5)
Systolic BP (mmHg), mean (SD)	135 (17)	133 (15)
Diastolic BP (mmHg), mean (SD)	77 (10)	76 (9)
LDL cholesterol (mg/dl), mean (SD)	78.7 (31.0)^e^	70.4 (27.7)^c^
HDL cholesterol (mg/dl), mean (SD)	44.8 (12.3)^e^	43.9 (9.4)^c^
Total cholesterol (mg/dl), mean (SD)	157.8 (40.2)^c^	142.4 (32.6)
Triglycerides (mg/dl), mean (SD)	187.7 (110.0)^d^	167.4 (119.0)^b^
Duration of diabetes (years), mean (SD)	14.7 (8.2)	15.3 (8.4)
Creatinine-based eGFR (ml/min/1.73 m^2^), mean (SD)	51.1 (10.4)	53.5 (9.9)
Cystatin C-based eGFR (ml/min/1.73 m^2^), mean (SD)	42.3 (12.3)	43.7 (13.6)
UACR (mg/g), median (IQR)	187.3 (60.5–546.4)	150.2 (54.4–464.2)
Previous CVD, *n* (%)	37 (34.9)	11 (33.3)
Heart failure (NYHA class I or II), *n* (%)	6 (5.7)	4 (12.1)
RAS inhibitor use, *n* (%)	104 (98.1)	33 (100)
ACE inhibitor use, *n* (%)	54 (50.9)	17 (51.5)
ARB use, *n* (%)	51 (48.1)	16 (48.5)
Other cardiovascular-related medication, *n* (%)		
Beta blockers	40 (37.7)	9 (27.3)
Calcium channel blockers	51 (48.1)	16 (48.5)
Diuretics	43 (40.6)	12 (36.4)
MRAs^a^	6 (5.7)	2 (6.1)
Glucose-lowering medication, *n* (%)		
Metformin	71 (67.0)	28 (84.8)
SGLT2i	41 (38.7)	19 (57.6)
Insulins	59 (55.7)	18 (54.5)
Sulphonylureas	19 (17.9)	3 (9.1)
DPP-4 inhibitors	18 (17.0)	4 (12.1)
Pioglitazone	3 (2.8)	2 (6.1)
Lipid-modifying agents, *n* (%)		
Statins	80 (75.5)	29 (87.9)
Other lipid-modifying agents	15 (14.2)	5 (15.2)

a Finerenone use was not permitted in the REMODEL trial.

b Data missing for one participant.

c Data missing for two participants.

d Data missing for three participants.

e Data missing for four participants.

ACE: angiotensin-converting enzyme; ARB: angiotensin receptor blocker; BP: blood pressure; CVD; cardiovascular disease; DPP-4: dipeptidyl peptidase 4; HDL; high-density lipoprotein; LDL; low-density lipoprotein; NYHA: New York Heart Association.

Both creatinine-based and cystatin C–based eGFR were calculated per the CKD-EPI 2009 equation [40].

**Figure 3:**
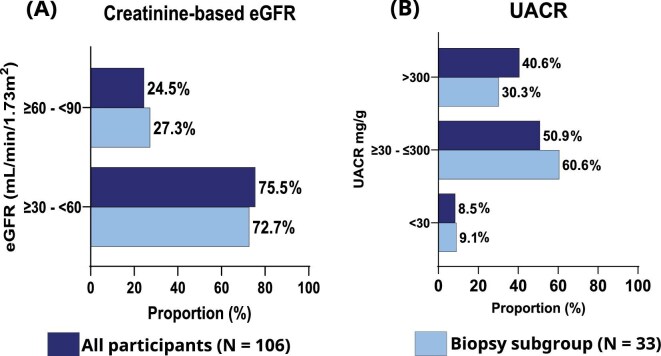
Kidney function assessments. **(A)** Baseline creatinine-based eGFR intervals in the full population and biopsy subgroup. **(B)** Baseline distribution of UACR in the full population and biopsy subgroup.

The mean baseline creatinine-based eGFR for the full population was 51.1 ml/min/1.73 m^2^ (SD 10.4) and 53.5 ml/min/1.73 m^2^ (SD 9.9) for the biopsy subgroup and the cystatin C–based eGFR was 42.3 ml/min/1.73 m^2^ (SD 12.3) and 43.7 ml/min/1.73 m^2^ (SD 13.6), respectively. The majority of both the full population and the biopsy subgroup had an eGFR of 30–60 ml/min/1.73 m^2^ irrespective of whether it was creatinine or cystatin C based (Fig. 3). The median urine albumin:creatinine ratio (UACR) was 187.3 mg/g [interquartile range (IQR) 60.5–546.4] and 150.2 mg/g (IQR 54.4–464.2) for all participants and the biopsy subgroup, respectively. Most participants for both the full population and the biopsy subgroup had a UACR of ≥30–≤300 mg/g (Fig. 3).

Overall, 98.1% of the full population and 100% of the biopsy subgroup were receiving RAS inhibitors at baseline. The most frequently used glucose-lowering medication at baseline was metformin, taken by 67.0% of the full population and 84.8% of the biopsy subgroup. SGLT2is were used by 38.7% and 57.6% in the full population and biopsy subgroup, respectively. Insulins (including premix) were used by 55.7% and 54.5% of the full population and biopsy subgroup, respectively. The most frequently prescribed lipid-lowering agents were statins, with 75.5% and 87.9% of the full population and biopsy subgroup, respectively.

**Figure 4:**
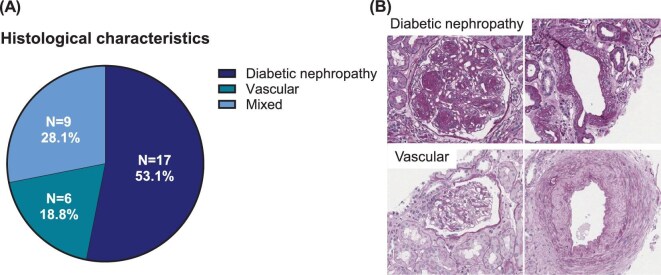
Histopathological analysis of biopsy subgroup samples from the REMODEL trial. **(A)** Proportional histopathological predominant characteristics. **(B)** Representative images of diabetic nephropathy and vascular biopsy samples. *One biopsy was diagnosed as membranous nephropathy and was therefore excluded from this analysis.

One kidney biopsy was diagnosed as membranous nephropathy and excluded from further analysis. Histopathological examination of the remaining biopsies revealed average global glomerulosclerosis of 27.9% (SD 21.8) and average interstitial fibrosis and tubular atrophy of 21.6% (SD 21.5). Glomerular tuft average area was 23 641 μm^2^ (SD 6803), the average mesangial index was 19.6% (SD 7.8) and the average GBM thickness was 685 nm (SD 195) (Table 3; individual analyses for each biopsy sample are presented in Supplementary Table 2). Within these 32 biopsies, three distinct endophenotypes emerged (Fig. 4) [37]. A predominantly classic diabetic nephropathy endophenotype (*n* = 17) displayed glomerulomegaly, mesangial expansion by matrix (diffuse or nodular) and significant thickening of the GBM, but none to mild arteriosclerosis. This group displayed a mean 23.2% (SD 19.8) global glomerulosclerosis, 15.2% (SD 14.3) interstitial fibrosis and tubular atrophy, a glomerular tuft area of 26 227 μm^2^ (SD 6383), a mesangial index of 21.0% (SD 9.8) and a GBM of 705 nm (SD 187) (Table 3). A predominantly vascular endophenotype (*n* = 6), such as is found in hypertensive nephropathy, lacked mesangial expansion but displayed moderate to severe arteriosclerosis, ischaemic-appearing glomeruli and more global glomerulosclerosis and tubulointerstitial scarring. The vascular endophenotype group displayed 41.5% (SD 29.7) global glomerulosclerosis, 27.8% (SD 35.4) interstitial fibrosis and tubular atrophy, a glomerular tuft area of 20 608 μm^2^ (SD 7093), a mesangial index of 16.7% (SD 3.2) and a GBM of 528 nm (SD 129). Finally, a mixed endophenotype (*n* = 9) showed morphologic features of both diabetic nephropathy and vascular features with moderate to severe arteriosclerosis. This group displayed 27.6% (SD 18.2) global glomerulosclerosis, 29.4% (SD 20.4%) interstitial fibrosis and tubular atrophy, a glomerular tuft area of 22 503 μm^2^ (SD 6687), a mesangial index of 19.1% (SD 6.1) and a GBM of 756 nm (SD 213). On follow-up baseline biopsy from one participant in the mixed endophenotype group, electron microscopy revealed paramesangial immune complex–type electron-dense deposits not previously seen (no glomeruli were available for ultrastructural examination in the entry biopsy) and possibly represent immunoglobulin A deposits. This biopsy was not excluded because clear features of diabetic nephropathy (e.g. GBM thickening) were observed. Seven biopsies showed focal segmental glomerulosclerosis, three in both the diabetic nephropathy and the mixed endophenotypes and one in the vascular endophenotype group.

**Table 3:** Summarized kidney histopathological characteristics based on the predominant endophenotype in the biopsy subgroup (*n* = 32^a^).

Predominant histopathological characteristic	Global glomerulosclerosis (%), mean (SD)	Interstitial fibrosis and tubular atrophy (%), mean (SD)	Glomerular tuft area (µm^2^), mean (SD)	Mesangial index (%), mean (SD)	GBM thickness (nm), mean (SD)	Arteriosclerosis (luminal area narrowing), %^b^
Diabetic nephropathy (N = 17; 53.1%)	23.2 (19.8)	15.2 (14.3)	26 227 (6383)	21.0 (9.8)	705 (187)	<25
Vascular (N = 6; 18.8%)	41.5 (29.7)	27.8 (35.4)	20 608 (7093)	16.7 (3.2)	528 (129)	>25^c^
Mixed (N = 9; 28.1%)	27.6 (18.2)	29.4 (20.4)	22 503 (6687)	19.1 (6.1)	756 (213)	>25^c^
Total average (N = 32^a^)	27.9 (21.8)	21.6 (21.5)	23 641 (6803)	19.6 (7.8)	685 (195)	n/a

a One biopsy was diagnosed as membranous nephropathy and was therefore excluded from this analysis.

b Arteriosclerosis (luminal area narrowing by vascular fibrous intimal thickening) scored according to the 2018 Banff classification [36].

c All but one biopsy displaying >25% luminal area narrowing.

Data were missing for some of the participants (see individual values in Supplementary Table 2).

## DISCUSSION

The REMODEL trial aims to elucidate the potential MoA of the kidney-protective effects of semaglutide in people with T2D and CKD, building on the clinical benefits demonstrated by the recently published FLOW trial results [13]. REMODEL is the first MoA trial for an incretin-based therapy in people living with T2D and CKD. Similar studies have now been initiated and include TREASURE-CKD (NCT05536804), T1-DISCO (NCT05819138) and REMODEL-T1D (NCT05822609). REMODEL employs a multifaceted approach, integrating advanced multiparametric MRI and clinical assessments and uniquely incorporates kidney tissue analysis through acquisition of biopsies. This distinctive approach combines advanced imaging techniques with state-of-the-art transcriptomic and morphometric analyses of kidney tissue samples from a subset of participants. The depth and breadth of data collected promise to significantly advance our understanding of semaglutide treatment effects on the kidney.

Deciphering the precise MoA of GLP-1RAs in T2D and CKD could improve treatment approaches, enabling precision medicine by identifying CKD subtypes with varying responsiveness to semaglutide and contextualize the findings in the FLOW trial [13]. This comprehensive mechanistic understanding may guide the development of combination therapies that address multiple pathways simultaneously, since in-depth MoA studies can facilitate insights into how to best combine pharmaceutical classes, e.g. those that have haemodynamic effects, which may be considered when initiating multiple drug classes with similar effects. Moreover, the wealth of information gathered may have implications beyond CKD, possibly informing other related conditions within CKM syndrome with or without T2D.

To maximize the application and reach of the REMODEL trial, a representative population of people with T2D and CKD has been enrolled. Most participants from the full population (>75%) had moderately impaired kidney function (eGFR ≥30–<60 ml/min/1.73 m^2^) and in general had a lower baseline UACR than in similar trials, including FLOW [41]. Participants receive a high standard of care, reflected in their concomitant medication use (e.g. metformin, RAS inhibitors and SGLT2i treatment). Perhaps consequently, glycaemic control levels in study participants were close to guideline-recommended levels (mean HbA1c of 7.1% and ≈45% with an HbA1c <7%), despite long (≈15 years) T2D duration [42, 43]. Participant characteristics are generally consistent between the full population and the biopsy subgroup, except for the higher use of metformin, SGLT2is and statins in the latter. Nonetheless, the overall similarities between the full population and the biopsy subgroup will support meaningful analyses.

As in previously reported kidney biopsy studies of people with CKD in T2D [37, 44, 45], different structural endophenotypes of CKD beyond classic diabetic nephropathy were observed in the REMODEL trial and, as such, the findings will be applicable to a broader population. Approximately half of the biopsy subgroup participants (53.1%) included in morphological analysis in the REMODEL trial had predominantly classic diabetic nephropathy features, including expansion of the mesangium by matrix, appearing nodular in some cases and clinically significant GBM thickening. Additionally, biopsies from 27.3% of participants displayed a mixed vascular and diabetic nephropathy phenotype, and 18.2% exhibited predominantly vascular endophenotypes, which could support analysis of the benefits of semaglutide that extend beyond classic diabetic kidney disease pathology. Mapping the imaging and tissue neighbourhood–anchored molecular profiling studies to the structural endophenotypes will help establish links between semaglutide-affected molecular pathways and changes in kidney structure and function, with the potential to extend these insights from patients with T2D and CKD to other CKD populations.

In conclusion, REMODEL's innovative multimodal approach, particularly its inclusion of multiparametric MRI and research kidney biopsies, provides an understanding of semaglutide's kidney-specific MoA in people with T2D and CKD. By enrolling a representative population with T2D and CKD, the trial ensures the generalizability and clinical relevance of the findings, with results expected in late 2025.

## Supplementary Material

gfaf114_Supplemental_File

## Data Availability

Data are available from the corresponding author upon reasonable request.
