# Supplementary material for: Rationale, design and baseline characteristics of REMODEL, a mechanism-of-action trial with semaglutide in people with type 2 diabetes and chronic kidney disease
Source: Nephrol Dial Transplant. 2025 Jul 3;40(11):2182–92. doi: 10.1093/ndt/gfaf114 (PMC12559791; doi:10.1093/ndt/gfaf114)
Supplement: gfaf114_Supplemental_File [file gfaf114_supplemental_file.docx]

| **Source of biomarker** | **Biomarkers** |
| --- | --- |
| Serum/plasma | HbA_1c_ |
|  | High-sensitivity C-reactive Protein |
|  | Interleukin 6 |
|  | Soluble Tumor Necrosis Factor Receptor-1 |
|  | Soluble Tumor Necrosis Factor Receptor-2 |
|  | Tumor Necrosis Factor Alpha |
|  | Uric Acid |
|  | Fibrinogen |
|  | Lipid-profile |
|  | Electrolytes |
| Spot urine | Monocyte Chemoattractant Protein-1 |
|  | Kidney Injury Molecule-1 |
|  | Neutrophil Gelatinase-associated Lipocalin |
|  | F2-isoprostanes per unit Creatinine |
|  | 8-oxo-2’-deoxyguanosine |
|  | Electrolytes |

Supplementary table 1. Blood and urine biomarkers to be assessed as exploratory endpoints in the REMODEL trial. HbA_1c_: Hemoglobin A_1c_.

| **Predominant histopathological characteristic** | **Global glomerulosclerosis (%)** | **Interstitial fibrosis and tubular atrophy (%)** | **Glomerular tuft area (µm^2^)** | **Mesangial index (%)** | **GBM thickness (nm)** | **Arteriosclerosis (luminal area narrowing) score^a^** |
| --- | --- | --- | --- | --- | --- | --- |
| Diabetic Nephropathy | 14.3 | 15 | **-** | 13.1 | 396 | 1 |
| Diabetic Nephropathy | 0.0 | 35 | **-** | **-** | 550 | **-** |
| Diabetic Nephropathy | 52.2 | 40 | 31,994 | 23.0 | 656 | 1 |
| Diabetic Nephropathy | 25.0 | 0 | 24,499 | 47.9 | 877 | 1 |
| Diabetic Nephropathy | 0.0 | 2 | 23,700 | 24.0 | 507 | 0 |
| Diabetic Nephropathy | 20.0 | 2 | 18,376 | 16.3 | **-** | 0 |
| Diabetic Nephropathy | 0.0 | 0 | 29,651 | 31.3 | 776 | 1 |
| Diabetic Nephropathy | 0.0 | 15 | 22,330 | 30.8 | 868 | 1 |
| Diabetic Nephropathy | 60.0 | 10 | 33,557 | 18.0 | 766 | 1 |
| Diabetic Nephropathy | 33.3 | 5 | 34,979 | 19.7 | **-** | 1 |
| Diabetic Nephropathy | 0.0 | 10 | **-** | 16.5 | 588 | **-** |
| Diabetic Nephropathy | 41.7 | 15 | 19,986 | 12.0 | 784 | 0 |
| Diabetic Nephropathy | 10.5 | 10 | 31,865 | 23.0 | 640 | 0 |
| Diabetic Nephropathy | 33.3 | 50 | **-** | 9.7 | 1,057 | 1 |
| Diabetic Nephropathy | 44.4 | 20 | **-** | **-** | 541 | 0 |
| Diabetic Nephropathy | 33.3 | 20 | **-** | 16.9 | 978 | **-** |
| Diabetic Nephropathy | 26.1 | 10 | 17,556 | 13.3 | 590 | 1 |
| Vascular | 42.9 | 30 | 15,521 | 13.1 | 442 | 3 |
| Vascular | 85.2 | 95 | 11,497 | 16.2 | 607 | 2 |
| Vascular | 0.0 | 2 | 21,765 | 15.0 | 533 | 3 |
| Vascular | 33.3 | 10 | 23,679 | 15.3 | 691 | 3 |
| Vascular | 62.5 | 30 | 19,184 | 18.8 | **-** | 1 |
| Vascular | 25.0 | 0 | 32,004 | 22.1 | 366 | 3 |
| Mixed diabetic nephropathy and vascular | 32.4 | 50 | 26,360 | 20.1 | 737 | 3 |
| Mixed diabetic nephropathy and vascular | 0.0 | 15 | 11,173 | 12.7 | 540 | 1 |
| Mixed diabetic nephropathy and vascular | 50.0 | 25 | 29,682 | 29.2 | 1,062 | 2 |
| Mixed diabetic nephropathy and vascular | 41.2 | 45 | 18,213 | 18.1 | 973 | 3 |
| Mixed diabetic nephropathy and vascular | 20.0 | 10 | 28,553 | 15.9 | **-** | 3 |
| Mixed diabetic nephropathy and vascular | 33.3 | 50 | 19,969 | 21.6 | 782 | 2 |
| Mixed diabetic nephropathy and vascular | 7.0 | 10 | 16,584 | 9.0 | 469 | 2 |
| Mixed diabetic nephropathy and vascular | 50.0 | 55 | 30,582 | 25.0 | 726 | 2 |
| Mixed diabetic nephropathy and vascular | 15.0 | 5 | 21,407 | 20.7 | **-** | 2 |
| Membranous nephropathy | 5.3 | 5 | 18,689 | 18.2 | **-** | 1 |

Supplementary table 2. Individual kidney histopathological characteristics based on the predominant endophenotype in the biopsy subgroup (n=33). ‘**–**‘ indicates that no analysis was performed due to technical constraints. ^a^Arteriosclerosis (luminal area narrowing by vascular fibrous intimal thickening) scored according to the 2018 Banff classification (37). GBM: glomerular basement membrane.
